# Why Prediction Matters in Multitasking and How Predictability Can Improve It

**DOI:** 10.3389/fpsyg.2017.02021

**Published:** 2017-11-22

**Authors:** Laura Broeker, Andrea Kiesel, Stefanie Aufschnaiter, Harald E. Ewolds, Robert Gaschler, Hilde Haider, Stefan Künzell, Markus Raab, Eva Röttger, Roland Thomaschke, Fang Zhao

**Affiliations:** ^1^Department of Performance Psychology, Institute of Psychology, German Sport University Cologne, Cologne, Germany; ^2^Institute of Psychology, University of Freiburg, Freiburg, Germany; ^3^Institute of Sports Science, Sports Centre, University of Augsburg, Augsburg, Germany; ^4^Institute of Psychology, Fern-Universität Hagen, Hagen, Germany; ^5^Department of Psychology, University of Cologne, Cologne, Germany; ^6^School of Applied Sciences, London South Bank University, London, United Kingdom

**Keywords:** prediction, predictability, multitasking, multitasking performance, sources of predictability

Prediction^1^ is an omnipresent principle of human behavior that can be fostered by predictability in the environment. We regard prediction as the mental representation of future event states or anticipated action consequences, and predictability as a property of certain events in the environment. On the assumption that predictability and prediction are beneficial for any kind of behavior, we argue that their benefits to relieving the human system are most evident when encountering *multiple* tasks. However, we predicate that their impact on multitasking is understudied and so we aim at dissociating prediction and predictability within multitasking contexts and at outlining different sources of predictability that have not been conflated under this term so far. From our opinion it follows that future multitasking research requires experimental designs and analyses that consider and unveil principles of prediction and the impact of predictability on multitasking performance.

## Omnipresence of prediction according to predictive coding principles

Blakemore et al. (2000) proposed that it is impossible to tickle oneself, because there is no difference between predicted sensory consequences of one's forward model and the actually experienced sensory consequences. This means that there is entirely no surprise, which is tantamount to a prediction error of zero. In a general sense, people predict the effect of an action without necessarily being aware of it (Wolpert et al., 2003), and, according to ideomotor theory, also initiate voluntary actions by the prediction of their effects.

Neurosciences have encouraged the idea of interpreting the cognitive system as a predictive coding machine, with the brain being seen as an anticipation device or feedforward processing machine (Bubic et al., 2010). Likewise, Friston (2010) has proposed the *free energy principle*, arguing that organisms try to counteract disorder by avoiding surprise (minimizing free energy). According to this principle, our internal states represent what has most likely caused a sensation, and we do not only try to evaluate these hypotheses in the external world, but permanently update them depending on the extent of the prediction error. The better the fit between internal and external state, the lower the free energy (Clark, 2013). The most important implication is that organisms have no need to search for regularities in the environment, but rather automatically adapt to them as a consequence of the continuous updating of representations about the external world due to the prediction error. Independent of specific models across disciplines, there is some agreement that these predictions are made outside of awareness, and we only become aware of them when they are violated and feelings of surprise draw attention to them (Whittlesea, 2004). However, it has been suggested that conscious predictions occur concurrently and independent from unaware predictions (Perruchet et al., 2006).

Accepting prediction as a permanently ongoing process of the cognitive and motor system, plus accepting multitasking as intrinsic part of both systems, implies that prediction should also leave its traces on multitasking. Multitasking paradigms, as typical testbeds for the capabilities and limits of motor-cognitive interaction, should therefore be eminently suitable to showcase that prediction and predictability matters for performance. For instance, prediction errors cannot decrease when different tasks are paired at random, when tasks are unpredictably sequenced or when different probabilities violate expectations about upcoming tasks. In contrast, predictability in task-environments, allowing people to develop predictions about tasks, stimuli or motor requirements, attenuate prediction errors, and ameliorate multitasking performance, which we will exemplify below.

## Sources of predictability in multitasking settings

Multitasking refers to task requirements in which cognitive processes involved in performing two (or more) tasks overlap in time, and it is typically investigated in dual-task or task-switching paradigms. Usually, multitasking is related to performance costs that manifest in increased reaction times for the second task or task switches and have for instance been explained by structural bottlenecks or the exhaustion of overall capacity limits (for overviews see Kiesel et al., 2010; Fischer and Plessow, 2015). Besides examining multitasking costs, one endeavor of multitasking research is the identification of sources fostering interference-reduction. We suggest that one major contributor to this is predictability in its various forms.

## Sequential structures

Dual-task studies suggest that people automatically use events (i.e., stimuli/responses) in one task to predict events in the other task (Jiménez and Méndez, 2001). While automatic prediction of elements in Task-A, based on elements in Task-B, disrupts sequence learning when one task is random, fast reactions and implicit sequence learning are preserved when stimuli and responses in both tasks are arranged such that predictive relationships between task elements hold (Keele et al., 2003; Röttger et al., 2017). In general, results from dual-task sequence learning studies suggest that prediction occurs automatically and is per default not depending on task boundaries.

Similarly, task switching studies suggest that people can acquire *implicit* knowledge about the sequences of *tasks* (Heuer et al., 2001; Koch, 2001). In task-switching setups where, unbeknownst to participants, tasks follow a regularly repeating sequence, participants respond faster as compared to baseline conditions where tasks switch randomly. Presumably, automatic prediction based on implicit task-sequence knowledge fosters the preparation of the upcoming task set.

## Time contingency

Other than enhancing predictability by structuring events or tasks, recent accounts have investigated the impact of interval durations between tasks, assuming that the temporal distribution of tasks may carry information about which task will occur. To investigate whether participants adapt to regularities of waiting time and task requirements, Aufschnaiter et al. (2017) employed a setting in which inter-task delays predicted the task type in the upcoming trial with different probabilities (70, 80, and 90%). Participants responded faster to frequent than to infrequent delay-task combinations for all tested degrees of predictability and both, task switches and repetitions, benefited from the predictability of time to task. Again, there is evidence for the omnipresence of prediction, as participants did not become aware of the predictive value of the interval duration.

## Explicit cues

In contrast to implicit predictability based on sequences or time, other studies manipulated predictability by providing *explicit* task cues that precede the imperative target stimuli (Meiran, 1996). Typically, these studies manipulate the duration of the interval between cue and imperative stimulus to investigate processes of task preparation (Kiesel et al., 2010), hypothesizing that longer preparation equals better prediction. They provide accumulative evidence that prolonging cue-stimulus intervals leads to reduced switch costs, indicating that switches benefit more from longer preparation times than repetitions (Logan and Bundesen, 2004), and that this effect is even more pronounced when additional cues are provided during preparation time (Koch, 2003).

Some studies also manipulated validity of explicit cues. In most studies, the cue predicts the task deterministically (100% valid), and any cue-based preparation will always be correct. However, in real life multitasking, cues often involve some degree of uncertainty, predicting tasks only probabilistically. Those few studies employing probabilistic cues observed preparation in terms of better performance for valid than invalid trials (Dreisbach et al., 2002; Wendt et al., 2012). Yet, results are inconclusive regarding preparation effects in switch and repetition trials (Dreisbach et al., 2002) vs. switch trials only (Wendt et al., 2012).

## Sensorimotor cues

In addition to external cues that precede the imperative stimulus, the system itself is capable of providing predictive sensorimotor cues *prior* to executing an action. Sensory signals and motor commands both provide useful information to reinforce internal forward models, capturing the causal link between actions and their sensory consequences. So, relevant sensorimotor cues would be internal predictions based on efference copies of motor commands (Synofzik et al., 2013). In dual-task tracking studies either the middle segment of the tracking path was repeated or participants were provided with visual guidance information. Both, implicit motor learning of the middle segment and the exploitation of visual information through feed forward control, improved tracking performance even in the presence of a secondary auditory detection task and independent of participants' awareness (Ewolds et al., submitted). Wolpert and Flanagan (2001) suggested that predictive control mechanisms can be best exploited when the environment is predictable, but that sensorimotor cues are the most useful signal for the system whenever the environment is unpredictable.

## Prediction in unpredictable contexts

In accordance with predictive coding, prediction is an ongoing process independent of predictability in the environment, and people seem to indeed predict upcoming trials even for random task and stimulus distributions. For instance, in sequence-learning experiments where tasks were drawn at random but participants were forced to predict the upcoming task, predicted tasks were performed faster than upcoming tasks not fulfilling the prediction (Gaschler et al., 2014). Likewise, task switching experiments revealed that participants respond faster to stimulus repetitions in repetition trials compared to repetitions in switch trials (Altmann, 2011). This effect has recently been ascribed to a “priming and inhibition” account (Druey, 2014), assuming that after response activation the corresponding response category is always inhibited, which leads to slower responses for repetitions in switch trials. Only if a stimulus in a specific task-set is repeated, there is an additional priming of the stimulus category, which outweighs the response inhibition, and enables participants to respond faster to stimulus repetitions than stimulus switches in repetition trials.

Furthermore, results of cued task-switching studies by Horoufchin et al. (2011a,b) demonstrate that participants predict repetitions of time structures in consecutive trials. The authors manipulated the duration of response-to-cue-intervals (RCI) in switch and repetition trials, and analyzed the effects of short and long RCI depending on whether they changed from the previous trial. They observed that participants responded faster in repetition trials compared to switch trials, and that this repetition advantage was of similar size for short and long RCIs when the RCI from the previous trial repeated. If, however, the RCI from the previous trial changed, the advantage of task repetitions decreased and switch costs increased. The overall results (especially the lack of RCI effects with unchanged RCI) were explained by a temporal distinctiveness account on episodic retrieval, which presumes that if a similar temporal relation between the previous and the current RCI exists, task episode of the previous trial can be retrieved in the current trial and repetition advantages unfold.

## Prospects

As outlined throughout the text, we presuppose automatic prediction of cues, tasks and stimuli or required responses, and hope to have convincingly conveyed that prediction and predictability matter for multitasking performance. Yet, multitasking studies often either ignore the impact of prediction or lack a measurement of it, and it is often hard to identify (and evaluate the role of) trials in which predictions mismatch the cue and the upcoming task. Thus, a consequence of this opinion would not only be the consideration of the system's predictive nature when conducting multitasking experiments, but the requirement to change analyses and research designs beyond core performance measures that capture prediction in multitasking behavior. We suggest that one way to realize this could be the implementation of more trial-wise analyses, because aggregating data over many trials might conceal the (incremental) impact of predictions. This might be especially important for settings that require learning or operate with predictions of varying validity. Further, reinforcing additional measures like error negativity/positivity would extend classic measures like reaction times (Alexander and Brown, 2011), which rather capture after-effects of valid or invalid predictions and do not adequately reflect core processing of prediction that occurs prior to stimulus presentation. Taking account of invalid predictions (e.g., by considering post-error slowing), would further lead to more nuanced understanding of performance differences. Other than that, it could be useful to consider people's awareness about predictions. Although we presuppose automatic prediction, there is evidence that making people realize the automaticity of predictions and actions may lead to deterioration of performance. For instance, Beilock et al. (2002) showed that multitasking performance in golf experts suffered when they had to pay attention to the step-by-step execution of putting, and attributed this to the intrusion into automatic processes that ground on well-developed forward models and predictions about future results of one's action.

## Author contributions

All authors listed have made a substantial, direct and intellectual contribution to the work, and approved it for publication.

### Conflict of interest statement

The authors declare that the research was conducted in the absence of any commercial or financial relationships that could be construed as a potential conflict of interest.

## Footnotes

^1^Prediction is equated with *anticipation* and *expectation* (Northoff, 2014, p. 146).
